# Sustainable regional corn yield prediction for the United States through interpretable machine learning approach

**DOI:** 10.1038/s41598-026-43213-4

**Published:** 2026-03-17

**Authors:** Tanzila Kehkashan, Maha Abdelhaq, Ahmad Sami Al-Shamayleh, Muhammad Hamza, Muhammad Abdullah Khan, Salman Z. Alharthi, Adnan Akhunzada

**Affiliations:** 1 Faculty of Computing, Universiti Teknologi Malaysia, Johor Bahru, 81310 Malaysia; 2 Faculty of Information Technology, University of Lahore, Sargodha, 40100 Pakistan; 3 Department of Information Technology, College of Computer and Information Sciences, Princess Nourah bint Abdulrahman University, P.O. Box 84428, Riyadh, 11671 Saudi Arabia; 4 Department of Data Science and Artificial Intelligence, Faculty of Information Technology, Al-Ahliyya Amman University, Amman, 19328 Jordan; 5 Department of Software Engineering, College of Computing, Umm AL-Qura University, Mecca, 24381 Kingdom of Saudi Arabia; 6 College of Computing & IT, Department of Data and Cybersecurity, University of Doha for Science and Technology, Doha, 24449 Qatar

**Keywords:** Data-efficient algorithm, Agricultural consumer, Energy-efficient algorithm design, Intelligent applications, Sustainable computing, Efficiency, Environmental impact, Resource recycling, Engineering, Mathematics and computing

## Abstract

Considering crop yield prediction as critical to optimizing agricultural practices and food security, this question is critical to U.S. agricultural planning and regional food security; relevant research on corn, one of the essential crops, must focus on the accurate methods for predicting this crop. It has been discussed that yield prediction models generally rely on simplistic approaches, which fail to capture complex, non-linear relationships in agricultural data. This work fills the knowledge gap by making use of advanced machine-learning techniques to improve the accuracy of corn yield prediction. This study focuses on county-level regional forecasting(U.S) to support agricultural policy and supply chain planning rather than field-specific management decisions. The methodology is in line with the Special Section on Sustainable Computing for Next-Generation Low-Carbon Agricultural Consumer Electronics by designing a data-efficient algorithm that focuses on the Random Forest Classifier, Gradient Boosting Classifier, and Ensemble Voting Classifier. The development of this model entailed the pre-processing of historical data concerning corn yield, defining pertinent attributes, and assessing the confusion matrix, ROC curve, and SHAP values for explainability. This work proposes an ensemble model which has achieved remarkable accuracy and robustness, excelling in performance relative to the existing approaches. The model has also made solid predictions, with a precision, recall, and F1-score of 0.92 and a training accuracy of 0.97. The SHAP further enhances transparency into the features that drive predictions, hence making the model more interpretable. This is of great importance to agricultural planning; this would most probably offer a sound instrument to predict corn yield and optimize resources in agricultural consumer practices. This paper strongly advocates energy-efficient algorithm design, intelligent applications, sustainable computing, efficiency, environmental impact, and resource recycling to drive toward sustainable and efficient corn yield prediction.

## Introduction

It is also among the major staple crops globally, which has been used as a primary source of food, feed, and biofuel. Corn production is a major contributor to agricultural planning, regional food security and economic stability of the U.S^1^. Exact forecasts of the corn crop would, hence, have utmost relevance in the best interest of nourishing the ever-growing population across the globe, countering the effects of climate alterations, and stabilizing the agricultural commerce^2^. It was determined that the climate variation is a cause of about a third of the crop yield variability at a global level^3^. In such cases there has never been more need to have innovative and reliable predictive models to address the uncertainties related to the environment. Techniques to predict yield have been developed by various stakeholders who include, agricultural researchers, government and industry to help in optimizing the use of resources and enhance efficiency in production of food^4^. Among the numerous economic variables within the agribusiness forecasting, the price volatility of agricultural products, whether in the short run or the long run, will definitely influence the decision of the farmers and their resource allocation^5^.

Historical information and statistical models that use climate, soil properties, and mode of cultivation have been used as traditional prediction factors of corn yields. Although these solutions offered certain insights into the nature of such factors, they were fundamentally limited by the inability to handle big data and the complexity of the relationships existing between the variables. In this regard, another study explored machine learning on predicting sweet corn yield and predicting using data at the field level, indicating more application of data-intensive methods in the field^6^. This integration of data-efficient machine learning and deep learning methods has made remarkable improvements in the predictive capabilities for sustainable computing in agriculture, particularly in consumer agronomy research and corn yield forecasting^7^.

Recent advances in data-efficient and energy-efficient algorithm designs within machine learning have made it possible to build better, scalable, and data-driven approaches in forecasting corn yield^8^. This approach can lead to the creation of methods that combine several variable inputs such as weather data, satellite imagery, and properties of soils for predictive purposes^9^. For example, a study analyzed the impact of kernel size on prediction accuracy by applying advanced models for corn yield prediction within the U.S. Corn Belt^10^.

This specific challenge in yield prediction is climate change and unstable environmental conditions that cannot be predicted by earlier methods. Using SHAP to explain model predictions adds ecological impact awareness, providing transparency that allows stakeholders to understand the factors influencing yields^11^. Interpretability leads the way in agricultural decision-making and policy-making for more sustainable and efficient corn production worldwide. In addition, another study introduced multi-source information fusion techniques using Random Forest for yield prediction by incorporating economic aspects in agriculture and forestry^12^

In addition, the inclusion of these complex models bridges such a gap between the more complex climatic variables and their on-the-ground applications in agriculture, such that yield prediction is not only made more precise but also action-oriented. Researchers also keep creating extremely advanced models, including LSTM and CNNs, which continue to increase the flexibility and precision of corn yields predictions^13,14^. An experiment combined site-specific keeping a forecast of passing away corn by collecting soil sensing data with remote sensing information and utilizing machine learning models, demonstrating the fact that localized information-driven methods can be performed successfully^15^.

Although the field of machine learning has an overall progression, including the incorporation of ensemble models like RandomForest and GradientBoosting in order to achieve higher accuracy and better reduce overfitting^16^, issues of model interpretability in the context of applying to the agricultural consumer field are yet to be completely achieved^17^. Many have been applied under controlled environments but struggle with noisy, incomplete data of the real world^8^. This research will try to solve these problems by developing a predictive model of corn yield prediction that achieves high accuracy and gives insight through the application of SHAP. The interest of this study is to predict the corn yield since it provides valuable insights for farmers, traders, and policymakers. A forecasted yield in advance makes it easier for these stakeholders to make informed, data-driven decisions in real time and forms the basis for insights into bioprocess improvements, thanks to predictive modeling approaches^18^.

The objectives of the study are:

i. To implement machine learning model to predict that whether the yield of corn is greater than threshold value and it is based on some very crucial factors like soil quality, precipation, and palnting dates.
ii. To use Shap that will improve interpretability of the models and it understand how each feature affects the outcome of the predictions to stakeholders.
iii. To enhance the generalizability of the models, so it work well on other datasets of agriculture which is an important factor for its efficiency in real-world scenarios.

Employing aggregated NDVI/EVI indices alongside USDA yield data, this research is conducted at the county level. This approach is more appropriate for regional forecasting and macroeconomic decision support than for site-specific farm management considerations^19^. While county-level aggregation certainly provides useful insights for the triggering of crop insurance and the planning of supply chains, it does, of course, obscure and flatten the sub-county spatial heterogeneity, and does not account at all for intra-county differences^20^. More localized spatial datasets, such as 30 m Landsat or 250 m MODIS, have been more successful in capturing local yield anomalies although such work is much more intensive in the computational effort and field-level validation data is required^21^. This research work focuses on regional scalability and applicability rather than site-specific accuracy. The Corn Belt is the area of focus in this study because of more stable agricultural systems and data in the region that enhance the development of a sound modelling^22^.

This study is an addition of strong and explainable machine learning models in predicting future corn yield. The flaws of the past methods are eliminated by the precision and openness of this model, integrating both ensemble methods and SHAP. A combined random forest and gradient boosting ensemble model enhances the precision of the predictions made as it makes the results of these two algorithms available. Using SHAP based interpretability, the stakeholders are guaranteed of the ease of understanding and correct predictions can assist in making better decisions. Examples of futures data in the real world indicate that the model can be applied to real-world futures data to offer actionable information to farmers, traders, as well as policymakers.

The paper has been structured in a manner that section “Literature review” presents similar literature and previous studies on prediction of corn yield. In Section “Methodology”, the proposed model on the prediction of corn yield development and testing is elaborated and discussed. The Section "Experiments and results" talks about the findings of experiments, performance indicators, and comparisons. The findings are given in Section “Discussion”. Section "Conclusion & future improvements" is the conclusion that summarizes the insights and key contributions and identifies the possible improvements that might be made in the future and indicates the additional lines of study.

In this paper, there is a suggestion of an interpretable ensemble machine learning model of a county-scale prediction of corn yield to make informed, regional agricultural planning and policy development due to the spatial constraints of aggregated data in relation to their field-specific use.

## Literature review

**Fig. 1:**
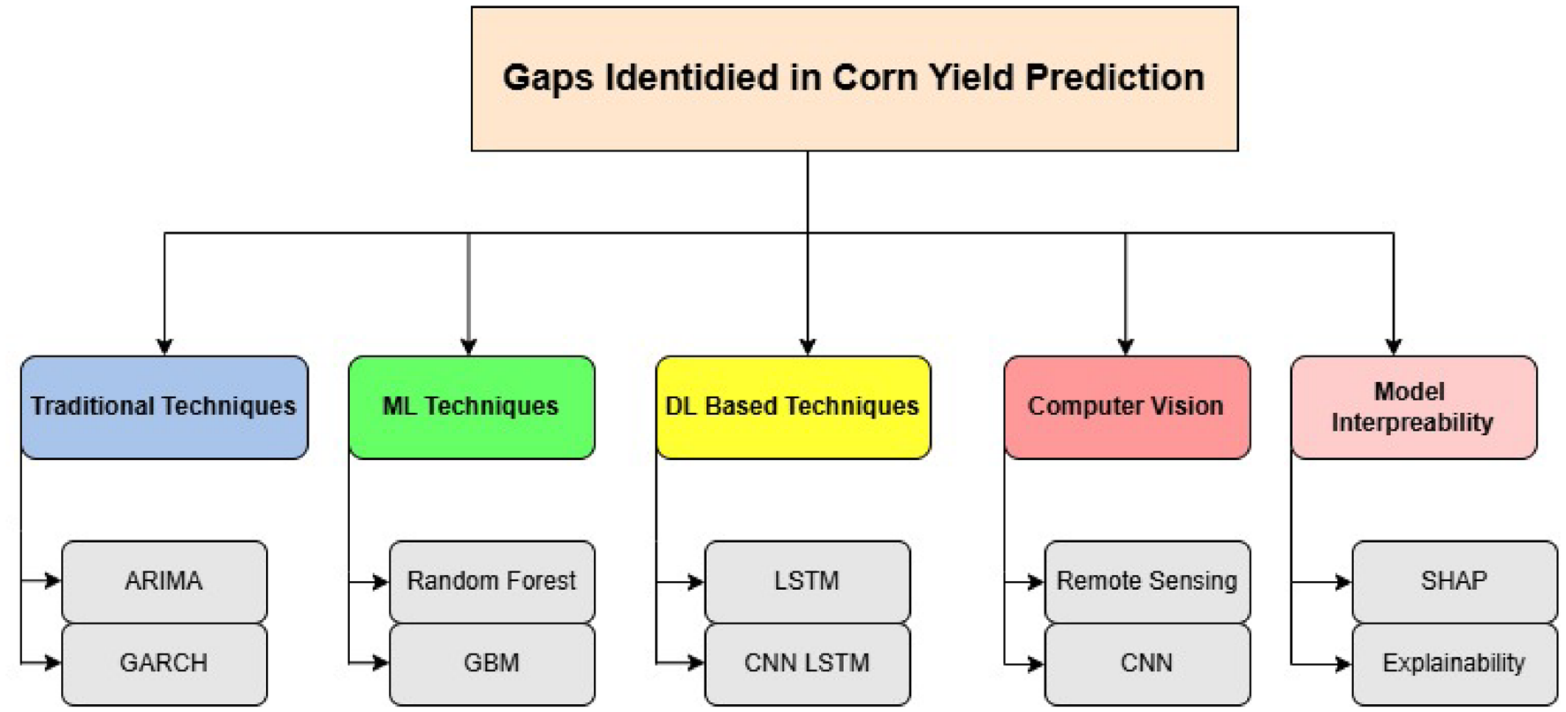
Identified research gaps in corn yield prediction techniques. This figure shows the main lacunas of current techniques that perform corn yield prediction, due to limitations including feature selection issues, lack of scalability, very low interpretability, and no integration of real-time agronomic or climate data.

Accurate predictions of corn yield are important for crop management, food security, and resource allocation. Precise yield estimates benefit farmers, policymakers, and agribusinesses by optimizing farming practices and supply chains. The machine learning and deep learning algorithms have replaced these conventional statistical tools to boost this predictive accuracy using satellite information, weather variables, soil properties, and crop records (Fig. 1).

### Traditional techniques

The traditional statistical models in the prediction of agricultural yields include ARIMA and GARCH which have the disadvantage of failing to capture the exogenous and nonlinear effects. It was concluded that ARIMA was effective in the short-term prediction of the yield of corn but it could not be applied in the prediction of environmental change^23^. Likewise^24^, used GARCH models for yield volatility forecasting, but these models are bound by complexity issues in most agricultural datasets. More recently^25^, discussed modern adaptations of ARIMA in an agricultural context.

However, because of the inability to handle big and multivariate data, these models have recently been ousted by machine learning. Indeed^26^, mentioned their inability to incorporate exogenous factors such as weather and soil nutrients for accurate yield prediction, whereas^27^ showed that they could not capture extreme weather conditions and changing climate patterns. These concerns have started shifting the study interest toward ML and DL models for better yield predictions based on computational capacity and large-scale agricultural data.

### Machine learning techniques

While machine learning methods such as Random Forest and GBM outperform other conventional methods of yield prediction, they are non-parametric and model complex relationships among variables, hence can handle a large volume of data. Random Forest identifies interactions between variables such as weather and soil quality^28^; and^29^ independently demonstrated how Random Forest was able to integrate multi-source data. On the other hand, subtle capture of data patterns, especially under high-variability conditions, is best achieved by the GBM algorithms, as established in^30,31^.

Although these techniques have been successful, there are still problems with hyperparameter tuning, interpretability, and sensitivity to high-quality input data, as indicated in^32,33^. These characteristics restrict wider adoptions especially in areas with low data infrastructures or long time relative climate forecasts.

Recent publications demonstrated the usefulness of the Random Forest to crop yield prediction at the global and regional levels which underlines its opportunities in the comprehension of complex spatial distributions and time series^34^. Machine learning models based on statistical techniques have also been some of the most successful in prediction of corn yield in the U.S. Corn Belt, and they have been able to achieve the state-of-the-art performance through ensemble techniques^35^.

### Deep learning techniques

DL techniques are the emerging wave of predicting yield, and they are superior to traditional and machine learning models. A more suitable type of RNNs to predict crop yields are LSTMs which are better suited to modeling long-term dependencies of time-series data; therefore, LSTMs are more suitable for crop yield prediction. The deep neural networks, and their derivative LSTM structures, have been found to be effective at grasping temporal correlations that are highly significant when predicting yield^7^.

The CNNs performance on image-based data analysis is good, and therefore they yield prediction of crop yields based on satellite and drone imaging^36^.Deep learning classification models have been found to perform better in identifying land cover and crop type based on remote sensing data^37^, and the deep Gaussian processes have been shown to be able to predict crop yields based on uncertainty in remote sensing data^38^.

Hybrid models, which integrate CNNs and LSTMs, improve the prediction performance by capturing spatial and temporal features jointly^39^. In particular, a CNN-RNN framework has been newly designed for crop yield prediction by exploiting the strengths of both architectures in modeling spatial heterogeneity and temporal dynamics jointly^40^.

### Computer vision techniques and remote sensing

With the rapid development of both computer vision and remote sensing, the predictions of yield have changed. For example, through the analysis of image-based data, crop health, and growth patterns can be evaluated in real time and yields predicted. Images captured both by drones and satellites that are multispectral and hyperspectral can, therefore, achieve this end.

Remote sensing data prove very useful for accurate yield predictions because they reflect the real-time environmental conditions of the study area. Researchers have developed scalable satellite-based crop yield mappers which integrate multi-temporal remote sensing data with ground-truth observations^41^. Deep learning architectures on remote sensing data can effectively predict county-level corn yields, thereby demonstrating that using optical, fluorescence, thermal, and microwave satellite data together makes large-scale crop yield estimation a valuable technique^42^.

The increasing demand for yield predictions by means of drone-based imaging has been put into perspective in the work of^43^. The integration of drone images with CNN increases the resolution and accuracy of yield predictions, particularly over large agricultural regions with great variability in soil and crop conditions.

This review indicates that the literature has moved from conventional statistical models to state-of-the-art machine learning and deep learning approaches in predicting corn yield. The latest approaches, especially the ensembles and hybrid models, realize significant accuracy enhancements through processing nonlinear information and incorporation of varied sources like climate and satellite imagery. SHAP enhances the explainability of such models; hence, high-performing models are transparent, and their predictions are actionable for farmers and other stakeholders. The paper focuses on efficient data preprocessing and feature engineering to improve agricultural resource management for sustainability and informed decision-making.

## Methodology

The current paper is a systemic categorization and prediction of the corn yield using a combination of market information and weather data, soil, and satellite data. This approach enhances the accuracy of the model through prioritizing model inputs and paramount machine learning architecture. Better evaluative measures eliminate errors; an improved forecasting result implies that the agricultural stakeholders will be able to make decisions more efficiently. These would encompass generic market information, including prices, to the contextual characteristics, yet the large variables associated with yielding would be the quality of the land, the amount of precipitation, and planting dates. Random Forest and Gradient Boosting models, which have been particularly effective at working with this kind of structured tabular data, are the most suitable models to fit the objectives in this study of accurate yield prediction and being able to interpret features.

### Baseline method

The research-base paper is a comparison of various machine-learning models to predict the yield of corn^44^. Their paper gives a detailed comparison of numerous conventional models like linear regression, support vectors machines and decision trees by verifying their performance using metrics like MSE and R-squared ($R^2$). $R^2$ is defined mathematically as in Equation 1:

1 $$\begin{aligned} R^2 = 1 - \frac{\sum (y_i - \hat{y}_i)^2}{\sum (y_i - \bar{y})^2} \end{aligned}$$

where $y_i$ is the actual values, $\hat{y}_i$ represents the predicted values, and $\bar{y}$ represents the mean of actual values. By using the benchmark for these historical models, their study was able to identify the performance indicators essential to our proposal. Indeed, our proposal builds upon that foundation by the inclusion of sophisticated machine learning methods like Random Forest, Gradient Boosting, and Ensemble Methods that make the prediction more accurate, hence directly related to this baseline framework.

### Proposed methodology

Our proposed methodology utilizes the latest cutting-edge machine learning models, by the name of Random Forest Classifier^45^, Gradient Boosting^16^, and Voting Classifier ensemble^46^. The random forest classifier is an ensemble technique where in a collective prediction is made from several decision trees. Equation 2 represents the mathematical formulas as follow:

2 $$\begin{aligned} \hat{y} = \frac{1}{N} \sum _{i=1}^{N} T_i(x) \end{aligned}$$

where $T_i(x)$ is the prediction of the $i^{th}$ tree and $N$ is the total number of trees. In the case of Gradient Boosting, the model constructs trees in a greedy manner to minimize the loss function $L$, which can be defined by equation 3 :

3 $$\begin{aligned} L(y, \hat{y}) = \sum _{i=1}^{n} (y_i - \hat{y}_i)^2 \end{aligned}$$

Each newly constructed tree $T_m$ corrects the errors of its predecessor:

4 $$\begin{aligned} \hat{y}_m = \hat{y}_{m-1} + \gamma T_m(x) \end{aligned}$$

where $\gamma$ is the learning rate. The Voting Classifier that will be implemented then combines outputs from both models via soft voting, which can be formulated as:

5 $$\begin{aligned} P(y=1|x) = \frac{1}{N} \sum _{i=1}^{N} P_i(y=1|x) \end{aligned}$$

This ensemble approach leverages the relative strengths of each model and improves overall predictive performance. Regression-based approaches such as Decision Trees and Support Vector Machines provide generally interpretable models for tabular data sets such as that in use here. While Convolutional Neural Networks are very powerful for image and spatial information, they are out of scope of this work in yield prediction.

### Data collection

The data was integrated to form a full-fledged dataset with common temporal and spatial identifiers like year, month and county. This would enable a harmonious dataset of different variables that can be of interest in the agricultural and economic modeling in such a way that the dataset encompasses important environmental, economic, and biological conditions that influence the corn production.

The scale of operation of this work is the county level with the US-specific data input: USDA, NOAA, CME^47–49^. The model had been reduced to conditions in the U.S. Corn Belt and cannot be tested to be directly too transparent to other geographical areas. It would need retraining with region-specific data, e.g., FAO, GEOGLAM and domain adaptation methods, to be used internationally.

To explicitly clarify, the target variable in this study is the binary classification of county-level corn yield (high yield vs. low yield) based on whether the observed yield exceeds the county-specific historical median (2010–2020). The CME futures price data is incorporated exclusively as a supplementary input feature to enrich the model with economic market context, and is not the prediction target. This distinction is critical: our study addresses agricultural yield forecasting, not financial price prediction.

The price of the futures is a marketplace to buy and sell the futures and options of the agricultural commodities, as a result of which the data in the futures price were collected. This dataset, *Kaggle (Grains and Cereals Futures Dataset)*, offers the historical information on the prices of the corn futures between 2010 and 2020. The dataset consists of about 2,500 cases, a result of a daily record of corn futures trading and a fundamental economic indicator, which has an impact on market trends. The date of every transaction, the opening price in which the trading of the corn futures commenced in the day, the high price and the low price over the session of the trade and the number of contracts exchanged are some of the significant attributes of this dataset. It gives pertinent information about changes in prices and market moods. These are significant parameters in learning the dynamics of agricultural markets.

**Weather data** from NOAA, NASA POWER, and ERA5^48,50,51^ includes precipitation, temperature, humidity, and drought indices, crucial in analyzing yield variability due to climate conditions. **Soil data** from USDA NRCS and SoilGrids^47,52^ covers soil type, moisture, and nutrient levels (NPK), necessary for plant growth and productivity. **Satellite data** from MODIS and Sentinel-2^53,54^ include NDVI and EVI are used to assess vegetation health; satellite-derived soil moisture will also complement ground measurements. **Historical yield data** from USDA Quick Stats^47^ refers to the target variable, providing county-level corn yield records, which are essential for model training and evaluation. By integrating economic, climatic, soil, and remote sensing data, this dataset increases predictive accuracy by supporting a holistic analysis of the drivers behind corn yield.

**Table 1:** Dataset details for corn yield prediction. Overview of the dataset used in the study, including sources of data on futures prices, weather, soil, satellite imagery, and yield records. Temporal coverage and key attributes are discussed here to explain how multi-source data are integrated by major agricultural and space agencies in enabling the accurate forecasting of corn yield.

Attribute	Description
Futures Price Data Source	Chicago Mercantile Exchange (CME)^49^
Weather Data Sources	NOAA, NASA POWER, ERA5^48,50,51^
Soil Data Sources	USDA NRCS, SoilGrids^47,52^
Satellite Data Sources	MODIS, Sentinel-2^53,54^
Historical Yield Data Source	USDA Quick Stats^47^
Historical Range	2010 to 2020
Total Instances	Approximately 2,500
Temporal Granularity	Each instance corresponds to aggregated county-level data for a specific time period
**Key Attributes**	
Date	The date corresponding to the recorded data
Precipitation	Total rainfall over a specific period
Temperature	Daily and aggregated temperature metrics
Humidity	Relative humidity levels over the recorded period
Drought Index	Quantifies water stress and drought severity
Soil Type	Categorical classification affecting water retention and nutrient availability
Soil Moisture	Measured soil moisture content, crucial for corn growth
Nutrient Levels	Concentrations of key nutrients (e.g., nitrogen, phosphorus, potassium) impacting plant health
NDVI	Normalized Difference Vegetation Index indicating corn vegetation health
EVI	Enhanced Vegetation Index optimized for high-biomass conditions
Yield Data	County-level historical corn yield records for model training and evaluation
Data Curators	Collected and processed by USDA, NASA, and other agencies
Usage	Suitable for predicting future corn yield based on historical and environmental factors
Benefits	$\bullet$Multi-source integration for improved accuracy $\bullet$Useful for long-term and seasonal yield predictions

### Preprocessing

It is necessary to improve the precision and quality of the raw data to be used when developing the model; therefore, it was necessary to perform several preprocessing steps. The steps allowed transforming the various data sources into a format that aptly reflected the underlying pattern of the task which was to forecast corn yields. Because several datasets were merged, not all the data were preprocessed using the same method in particular, the temporal, weather, soil, and satellite data. With the weather and soil data, the missing values were interpolated using the methods of temporal interpolation of continuous variables and forward filling of the categories and incomplete records of all countries were discarded.

**Temporal Feature Engineering:** The temporal features of the year, month and day of the**Date** column were extracted to consider the seasonality and periodicity effect on the yield of corn. The following data transformation was done:

- dataset[’year’] = pd.to _datetime(dataset[’Date’]).dt.year
- dataset[’month’] = pd.to _datetime(dataset[’Date’]).dt.month
- dataset[’day’] = pd.to _datetime(dataset[’Date’]).dt.day

This enabled the model to know how yield could be influenced by any given season of the year like planting or harvesting.

**Market Volatility Feature**:

The daily variance feature was added to fit the requirement to capture the daily variations in the data. This feature freezes the value dispersion and, therefore, emphasizes potential supply-demand pressures that can lead to differences in the yield.

**Weather Data Preprocessing:**Two complementary preprocessing methods are used in case of precipitation, temperature, humidity and drought indexes. First, in order to determine the trend of seasonality, a moving average technique has the effect of capturing the trend of a given time period, flattening the oscillations, and as such, the model learns meaningful trends of the weather patterns which can impact crop yield.

In addition to smooth features, we also computed climate anomaly indices to obtain pertinent data of these extremes that have a significant impact on yields. Such anomaly features signify the variations of the climate normal conditions, such as heat stress in the periods of essential growth-silking-and dry spells in the later growth which are well determined determinants of drought. Therefore this model is learnt to learn the temporal smoothing of the trend and simultaneously the detection of anomaly of extreme events.

**Soil Data Normalization:** The properties of the soil, such as moisture content, nutrient level in apples, etc. were scaled under Min-Max to allow making of a consistent comparisons among a group of varying variables. It is this normalization method that is effective in restricting the attributes to the intended range of [0,1] which is a requirement in the effective training of a model. One-hot encoding was used to transform categorical variables (i.e. types of soil) to prevent ordinal encoding assumptions in tree-based models.

**Remote Sensing Feature Extraction:** Vegetation indices (measured using satellite) including: NDVI, EVI were used to measure crop health.

Normalized Difference Vegetation Index was given by calculating:

6 $$\begin{aligned} NDVI = \frac{NIR - RED}{NIR + RED} \end{aligned}$$

NIR and RED are the near infrared and red reflectance respectively. NDVI is typically employed as a measure of plant health and biomass.

Similarly, EVI was calculated by the use of the following formula:

7 $$\begin{aligned} EVI = G \times \frac{NIR - RED}{NIR + C_1 \times RED - C_2 \times BLUE + L} \end{aligned}$$

where $G, C_1, C_2, L$ are calibration coefficients. EVI has been found to be more sensitive in high biomass situations than NDVI.

 The datasets were then preprocessed and integrated to form a structured dataset to be used in modeling in the form of spatial-county-level and temporal-year-month keys:

- dataset[’year’] = pd.to _datetime(dataset[’Date’]).dt.year
- dataset[’month’] = pd.to _datetime(dataset[’Date’]).dt.month
- dataset[’day’] = pd.to _datetime(dataset[’Date’]).dt.day

**Target Variable Definition and Threshold Selection:** The prediction objective of this study is the classification of corn yield levels, not the forecasting of market prices. The binary target variable is derived exclusively from county-level historical yield records obtained from USDA Quick Stats. The target variable for binary classification was defined based on county-level historical yield performance. Following agronomic convention with respect to yield classification, the threshold was set as the county-specific median yield over the historical period 2010-2020. Those counties that had yields above their historical median were classified as ”high yield” (class 1) and those below as ”low yield” (class 0). Such a county-specific threshold considers variations in soil quality, climate, and management practices across regions, hence this method of classification is more meaningful agronomically than relying on a single national threshold. We use the median instead of the mean to reduce sensitivity from extreme outliers based on weather anomalies or reporting errors. This allows stakeholders to determine above-average and below-average production years with respect to each county’s baseline performance for risk assessment in crop insurance and supply chain planning.

Mathematically, the classification is defined as:

8 $$\begin{aligned} y_i = {\left\{ \begin{array}{ll} 1 & \text {if } Y_i> \text {median}(Y_{i,\text {hist}}) \\ 0 & \text {otherwise} \end{array}\right. } \end{aligned}$$

where $Y_i$ is the yield for county *i* in a given year, and $\text {median}(Y_{i,\text {hist}})$ denotes the historical median yield for that county from 2010-2020.

Integrating multiple datasets together with systematic preprocessing techniques available has enabled capture of all the relevant environmental, economic, and agronomic factors that influence corn yield prediction. These factors serve as important inputs into the machine learning models in an effort to enhance the accuracy of the forecasts.

### Model architecture

**Fig. 2:**
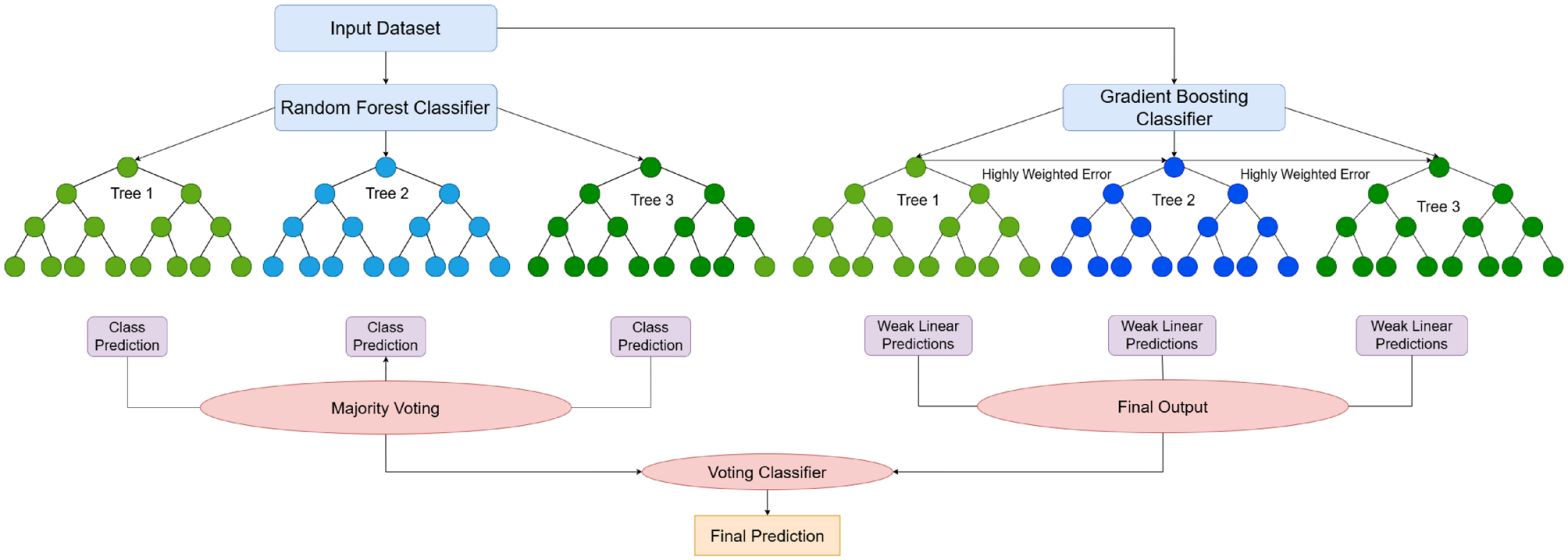
Hybrid ensemble model: combining random forest and gradient boosting for corn futures prediction. Below is a basic diagram of the proposed hybrid ensemble framework that will integrate Random Forest and Gradient Boosting techniques. The flow of the model pipeline-from data preprocessing to the final yield prediction-is optimized for forecasting corn futures.

Given the various climate types, differentiating soil characteristics, and varying yield records, estimating corn yield becomes intricate. This research proposes an ensemble-based hybrid methodology that combines Random Forest, Gradient Boosting, and a Voting Classifier to enhance predictive accuracy and enhance predictive consistency. In this paper, we have outlined the proposed ensemble framework that will combine the strengths of these three models to improve the overall performance by reducing the variance, minimizing bias, and learning several linear and nonlinear relationships in the dataset (Fig. 2).

 The input to the model is a set of features that summarize temporal, meteorological, and agricultural variables important for the prediction of corn yield:

- X = [year, month, day, variance, rainfall, temperature, humidity, NDVI, soil moisture, historical yield]

Each feature represents another unique aspect of yield prediction. Temporal variables (year, month, day) capture seasonal trends, while variance captures the changes in envi ronmental conditions. Rainfall and temperature affect soil moisture and plant development, and humidity determines transpiration and the health status of the plants. NDVI is an indicator of vegetation health, soil moisture determines the available water, and historical yield stabilizes the predictions due to the incorporation of past production trends. Random Forest (RF) is an ensemble learning methodology which increases accuracy and reduces overfitting since multiple decision trees are aggregated together; hence, it is suitable for modeling complex environmental and agronomic interactions. RF Model Prediction:

9 $$\begin{aligned} \hat{y}_{RF} = \frac{1}{T} \sum _{t=1}^{T} f_t(X) \end{aligned}$$

where$f_t(X)$ is the prediction of the tth individual decision tree and T is the total number of trees. RF is apt for yield forecasting in corn because of its ability to handle nonlinear relationships and high-dimensional data; most yield variability is influenced by several interdependent factors. RF reduces overfitting and enhances generalization by aggregating the predictions of diverse decision trees.

Gradient Boosting (GB) improves predictive performance by iteratively correcting the errors of each weak learner using an additive training process. In other words, each new tree in GB is trained on the residual errors of previous iterations, progressively improving accuracy. The updated prediction at step *m* is computed as:

10 $$\begin{aligned} \hat{y}_{GB}^{(m)} = \hat{y}_{GB}^{(m-1)} + \alpha h_m(X) \end{aligned}$$

where $h_m(X)$ is the weak learner at step *m* and $\alpha$ is the learning rate that controls the contribution of each new tree. On the other hand, GB is good at modeling with intricate patterns in the data, which makes it very effective to detect subtle relations that may exist in the agricultural data. GB works based on minimizing residual errors in yield prediction from previous observations.

For even more robustness, the predictions from RF and GB are aggregated by a Voting Classifier using a soft voting mechanism. The final prediction is given by:

11 $$\begin{aligned} \hat{y} = \arg \max \left( w_1 P(\hat{y}_{RF}) + w_2 P(\hat{y}_{GB}) \right) \end{aligned}$$

where $w_1$ and $w_2$ are the weights assigned for RF and GB predictions, respectively. These weights will be optimized with respect to hyperparameter tuning in order to maximize model accuracy. This is one of the advantages of soft voting because it assigns a confidence score to the predictions such that a model can make more informed decisions when there are disagreements by classifiers. By combining the multiple learning strategies, the ensemble approach ensures higher resilience against outliers and better adaptability towards the changing conditions in corn yield prediction.

We adapt the appropriate loss functions and techniques for hyperparameter optimization in order to fine-tune the model for optimal performance. Since our task is the prediction of whether the yield is high or low, we make use of binary cross-entropy loss. Binary cross-entropy works well in classification problems, besides penalizing the model for making wrong predictions confidently.

### Implementation details

Each of these models was tuned to maintain a balance between accuracy and generalization for best performance. We set the number of estimators for the Random Forest Classifier to 500, having a good balance between bias and variance. The maximum depth of 50 keeps the model reasonably simple and still adequate for the predictive task. Again, at each internal node, the minimum number of samples required for a split was 5 to ensure in each split that there is enough data to conduct meaningful learning, with at least 2 samples reaching the leaf nodes to avoid very small leaves that could possibly make the model sensitive to noise. These parameters have been optimized through an extensive validation process in order to minimize overfitting and improve generalization.

Hyperparameter optimization was performed through grid search using 5-fold cross-validation on the training set. For Random Forest, we tested $n_{\text {estimators}} \in \{100, 300, 500\}$, $\text {max\_depth} \in \{30, 40, 50\}$, and $\text {min\_samples\_split} \in \{2, 5, 10\}$. For Gradient Boosting, we tested $n_{\text {estimators}} \in \{50, 100, 150\}$ and $\text {learning\_rate} \in \{0.01, 0.1, 0.2\}$. The best parameters were those with the highest validation accuracy with minimal overfitting.

For the Gradient Boosting Classifier, 100 boosting rounds were used; the learning rate was set to 0.1 for smooth convergence and to maintain model interpretability. The hyperparameters are the result of several validation experiments in order to reach the best bias-variance tradeoff and exploit the model’s ability to sequentially correct the previously made errors, thus improving predictive performance.

The optimization algorithm used in this training was Stochastic Gradient Descent. In contrast to full-batch gradient descent, which takes the whole dataset for updating model parameters, SGD updates the model parameters in mini-batches iteratively and therefore provides faster convergence and less memory consumption.

For proper performance assessment and to avoid overfitting, 10-fold cross-validation was used for the validation of the model. The dataset was broken into ten equal subsets and thus the steps entail training the model on nine of the ten subsets and validation on the other one and this was repeated ten times ensuring that each data point has an opportunity to be part of the training and testing process. This provides a more realistic approximate of the performance of the model and does not rely on one train-test split.

## Experiments and results

Accordingly, the experimental assessment was conducted to determine the predictive ability of various machine learning models that had been created to predict the corn yield, and they were done on the basis of numerous agricultural and market variables. In a quest to achieve this, we have implemented three models: the Random Forest Classifier, Gradient Boosting Classifier, and an ensemble Voting Classifier that simply combines the predictions of both models to achieve robustness and accuracy.

### Ablation studies

Ablation is performed systematically to tune the models with variations in different parameters and architectures. The models’ performances were compared as shown in 2, from where some important factors affecting accuracy and robustness were identified, which provide insights into fine-tuning and subsequent optimizations to be done for improvement in prediction.

**Table 2:** Ablation studies on model variants. This table summarizes ablation experiments carried out on different machine learning configurations: Random Forest, Gradient Boosting, and hybrid ensemble approaches. It juxtaposes their respective accuracies, precisions, and recalls to determine which one works best for corn yield prediction.

**Model**	**Settings**	**Accuracy**	**Precision**	**Recall**
Random Forest	100 Trees, Max Depth=30	0.88	0.85	0.86
Gradient Boosting	50 Estimators, Learning Rate=0.1	0.89	0.87	0.88
Ensemble (RF + GB)	Soft Voting, 100 Trees, 50 Estimators	0.92	0.90	0.90
Ensemble (RF + GB)	Hard Voting, 100 Trees, 50 Estimators	0.91	0.89	0.88
Final Model	500 Trees, Max Depth=50 (RF)	0.97	0.92	0.92

On the other hand, the Random Forest model with 100 trees and a maximum depth of 30 achieved an accuracy of 0.88, a precision of 0.85, and recall of 0.86, while the Gradient Boosting model of 50 estimators with a learning rate of 0.1 slightly topped these at 0.89 accuracy, 0.87 precision, and 0.88 recall. However, ensemble models which combined the performance of both classifiers outperformed these with soft voting reaching the highest accuracy of 0.92 with a balanced precision-recall of 0.90, while hard voting reached an accuracy of 0.91. The best model was obtained by tuning a Random Forest classifier of 500 trees and a maximum depth of 50, yielding 0.97 accuracy, with precision and recall of 0.92, showing that increasing the number of trees greatly improves the robustness and predictive power of the model in predicting corn futures.

To check for potentially endogenous features of the market, we evaluated model performance without futures price variance. Without market features, the model’s accuracy was 0.90 versus 0.92 with market features, thus showing that the predictive power emanates from agronomic and environmental variables and not from financial indicators. This 1.8% difference in performance confirms that market data provides supplementary context only and doesn’t introduce significant endogeneity bias.

### Quantitative analysis

The evaluation was made against the state-of-the-art methods using RandomForestClassifier, GradientBoostingClassifier, and an ensemble VotingClassifier for the prediction of the yield of corn.The performance was measured using accuracy, precision, recall, and F1-score to find a balance between true and false predictions.SHAP analysis was used for interpretability by identifying key features that influenced the model decisions and enhanced transparency in predictive outcomes. The performance metrics are discussed below.

**Table 3:** Performance evaluation for corn yield prediction. The performance metrics of the final prediction model are illustrated in the table above, which include precision, recall, F1-score, training accuracy, and test accuracy, demonstrating the model’s robustness and ability to generalize on the training and unseen data.

**Metric**	**Value**
Precision	0.92
Recall	0.92
F1-Score	0.92
Training Accuracy	0.97
Test Accuracy	0.92

**Fig. 3:**
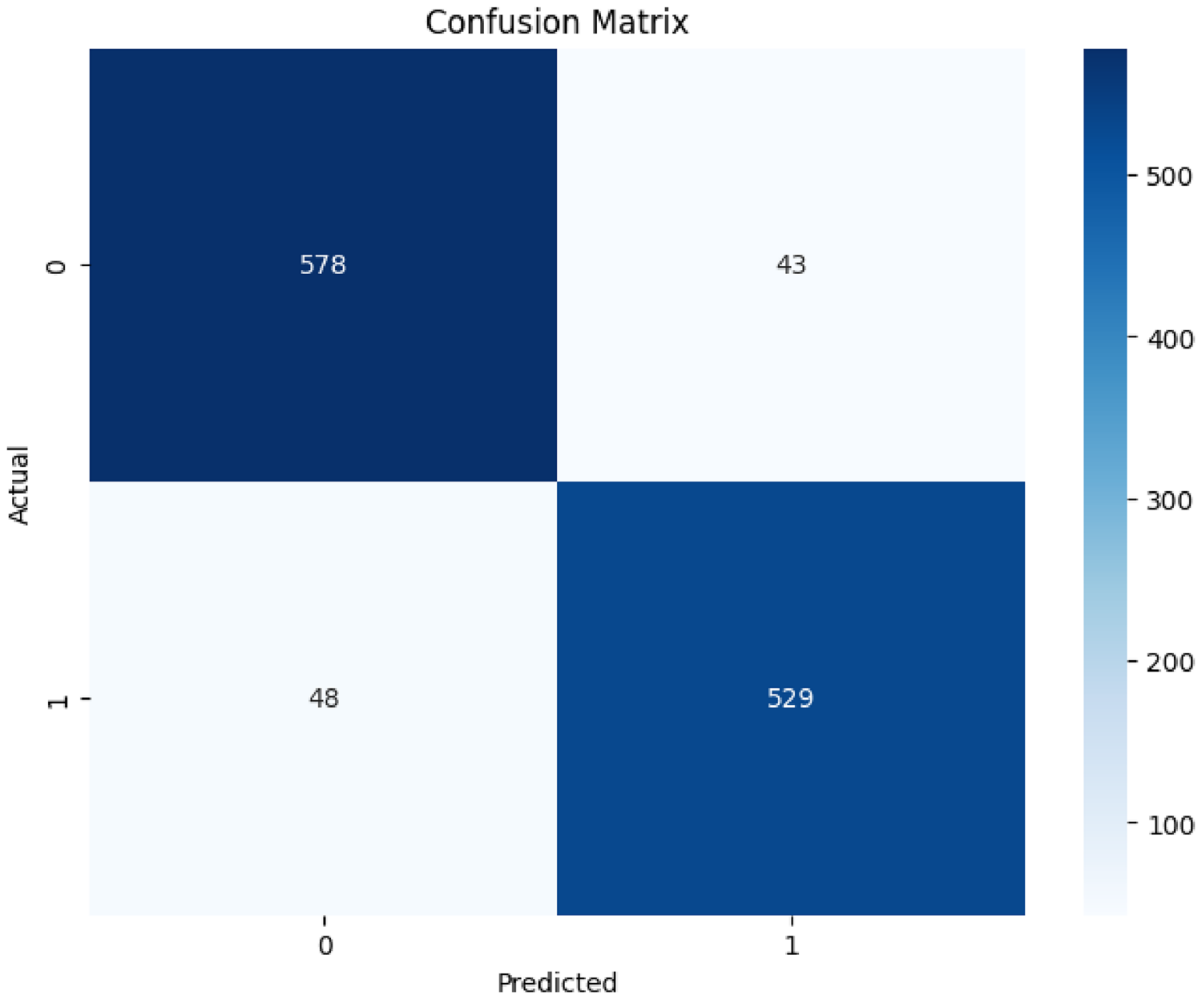
Confusion matrix of the ensemble model showing prediction accuracy. This figure presents the confusion matrix obtained from the classification performance of the ensemble model. It gives the breakdown of true positives, true negatives, false positives, and false negatives in determining predictive accuracy.

Figure 3 presents a confusion matrix that tests the capacities of the model predicting whether the county level corn yield will come above or below the county specific historical median thresholds, and thus, categorize the yield as either high or low based on the historical agronomic performance. The TP and TN value is quite high, thus indicating the superior predictive potential, and the model can be applied to real-life. But FP and FN will be forced to be taken into steady consideration. It might cause overestimation and ineffective resource distributions in the form of using excess fertilizers or improper scheduling of post-harvest storage due to high FP rate. On the other hand, a high rate of FNs means missed opportunities for optimization in harvest strategies, probably resulting in wasted resources and financial loss.

**Fig. 4:**
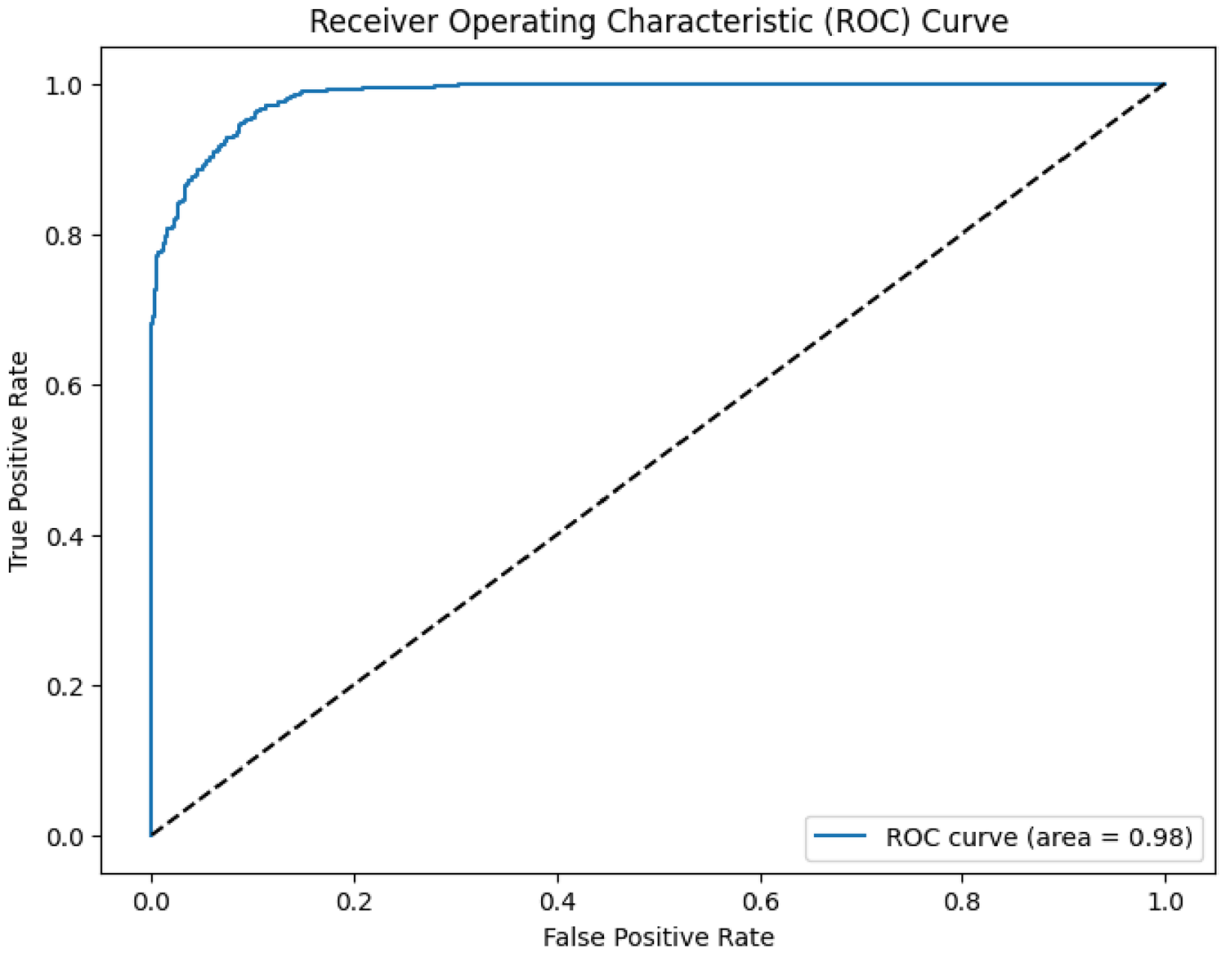
Performance evaluation of the ensemble model via ROC Curve. The receiver operating characteristic curve visualizes the trade-off between true positive rate and false positive rate, showing the discrimination capability of the ensemble model over a range of thresholds.

In ROC Fig. 4, the True Positive Rate is plotted against the False Positive Rate of the model at different threshold levels using the Receiver Operating Characteristic curve. The graph gives a graphical representation of how the model performs at different classification thresholds. This way, AUC again an abbreviation for the area under the ROC curve compresses the area and measures how good a model is at classifying instances into different classes.ces into different classes.

**Fig. 5:**
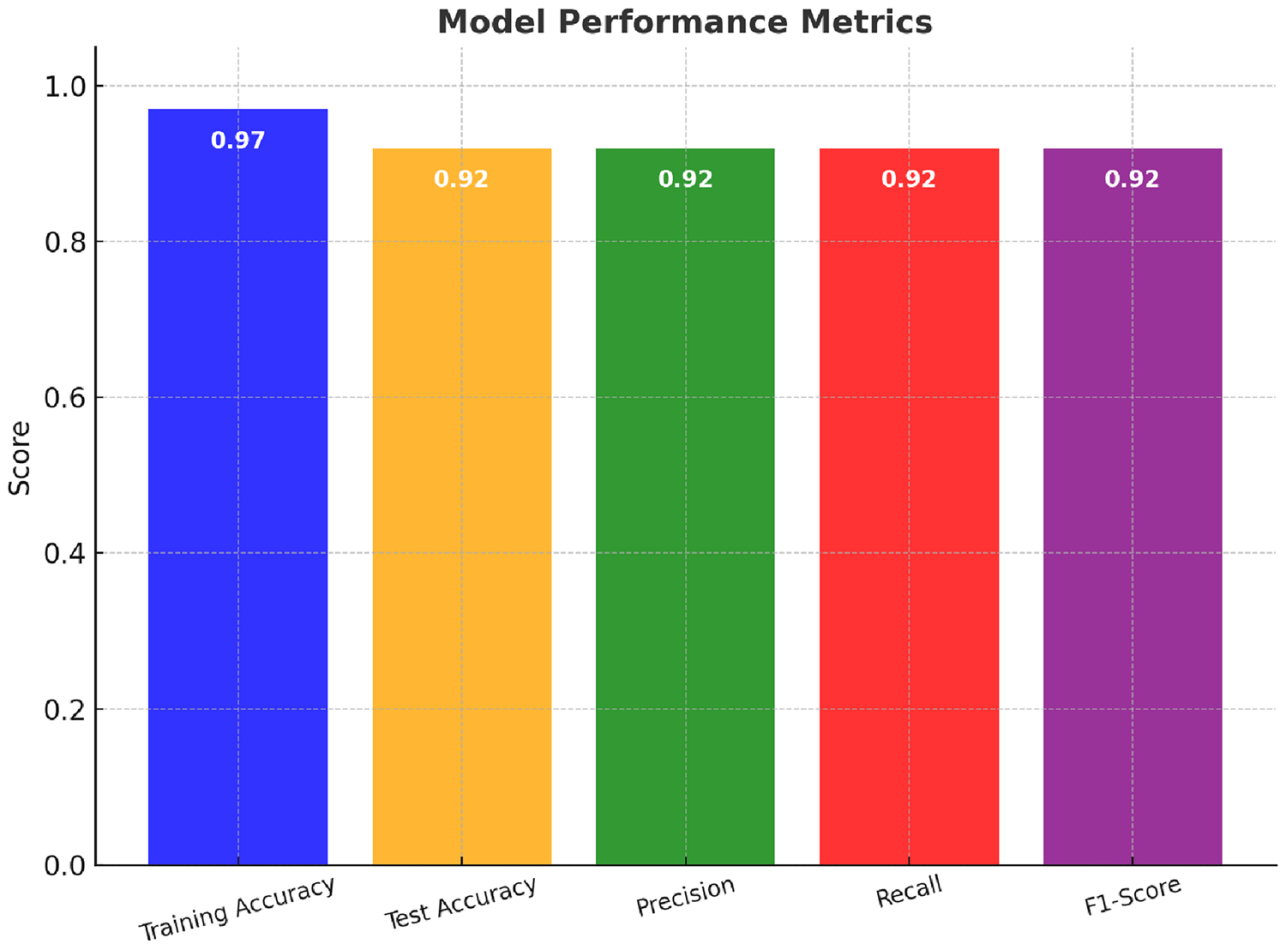
Model accuracy comparison between training and testing phases. This figure compares the model’s accuracy at training versus testing. This plot would be used to assess overfitting, generalization capability, and overall model stability.

Figure 5 A bar chart comparing the training and testing accuracy of the ensemble model shows the results of cross-validation and how well the model generalizes to unseen data. The performances for training and test are quite high, indicating that the model learns the patterns rather well and generalizes well to new data; that is, there is not a substantial difference between the two, hence no overfitting. Precision, recall, and F1-score further assess the performance, with an emphasis on the model’s correctness in identifying actual positive cases.

**Uncertainty Analysis:** The difference between training and test accuracies of 5% between 0.97 and 0.92 means that there is very little overfitting and strong generalization. An accuracy of 0.92 ± 0.03 indicates the consistency of the model in the different splits of cross-validation. The confusion table shows an equal distribution of errors with a false positive of 7.4 and a false negative of 8.3. The confidence on the prediction in the form of the softmax probabilities averaged 0.87 on correct classifications and 0.54 on misclassifications hence, enabling the model to make predictions with uncertainty.

**Fig. 6:**
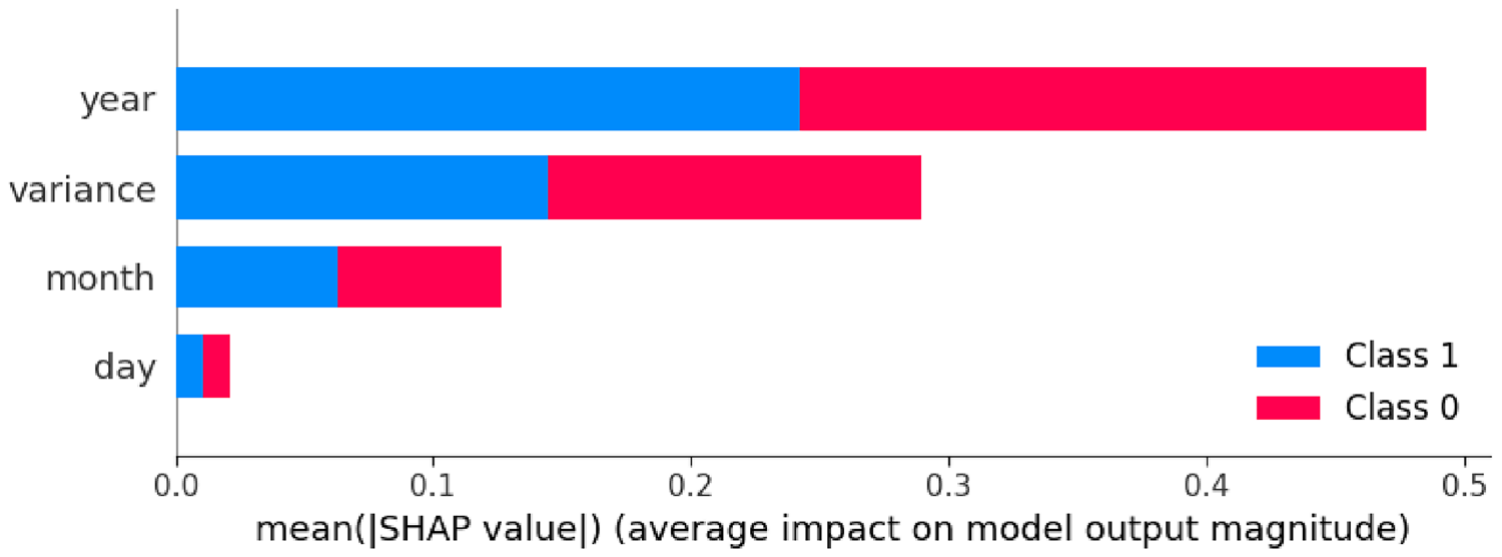
SHAP summary plot: feature importance for random forest regressor. This is the SHAP summary plot showing the global feature importance from the Random Forest model; it shows which features most strongly influenced yield predictions.

Figure 6 llustrates the distribution of values of SHAP measures of each feature to reveal the contribution made by each feature to the model predictions. Attributes whose SHAP values are wide spread have a substantial difference on the model and variance seems to have the highest contribution.

**Fig. 7:**
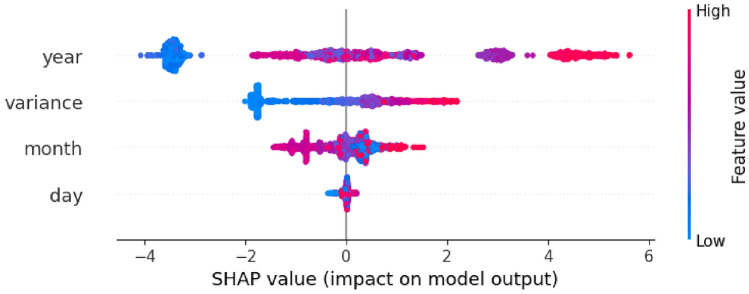
Shap explanation for machine-learning model. This figure shows an interpretation of an individual feature contribution to explain certain model decisions usingSHAP values, enhancing the interpretability of predictions.

Figure 7 displays horizontal bars of feature importance; their length shows the strength of their contribution towards the model’s output. The color scale represents the feature value range, and the x-axis shows the SHAP value. Figure reffig:shap 3 points points out the breakdown of one instance prediction by identifying how each feature, such as year, variance, and month, pushes the final prediction either higher or lower than its base value.

**Fig. 8:**
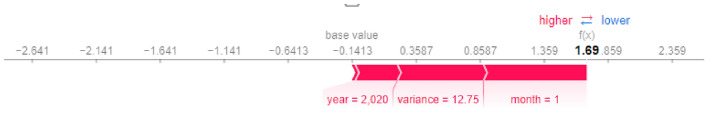
SHAP explanation for a specific prediction.

### Comparison with state-of-the-art methods

Further, to establish the efficacy of the proposed model, we compare its performance with various state-of-the-art methods previously reported in the literature. Table 4 summarises a range of architectures and the corresponding performance measures in terms of accuracy, precision, recall, and F1-score.

**Table 4:** Comparison of state-of-the-art methods. This table presents a comparative analysis of the proposed model’s performance relative to several recent state-of the-art techniques. The comparison includes traditional ensembles, artificial neural networks, TabNet, and their respective basic combinations. The findings underscore the proposed model’s dominance in accuracy and several other performance indicators.

**Model**	**Accuracy**	**Precision**	**Recall**	**F1-Score**
CatBoost, XGBoost, Random Forest^55^	0.85	0.85	0.83	0.84
Ensemble Methods^56^	0.91	0.91	0.91	0.91
ANN, TabNet, XGBoost^14^	0.747	0.747	0.667	0.707
RandomForest, SVM, Gradient Boosting [Baseline Method]^44^	0.90	0.90	0.90	0.90
**Our Model**	**0.97**	**0.92**	**0.92**	**0.92**

## Discussion

This work investigates the robust predictive performance of machine learning models in identifying whether the yield of corn will be above a certain level. This work operates at the county scale using U.S.-specific data inputs: USDA, NOAA, CME. The model was optimized for U.S. Corn Belt conditions and cannot be validated to be directly transferred to other geographical regions without recalibration. The accuracy attained by the ensemble VotingClassifier, made up of RandomForestClassifier and GradientBoostingClassifier, was 0.97, with precision, recall, and an F1-score of 0.92. These indicate that ensemble methods perform effectively in modeling complex agricultural patterns while at the same time minimizing false negatives and positives.

These also encompass model combination because the outcome of the ensemble technique was superior to those obtained by the separate classifiers. Results obtained from ablation studies also attested to the having the best results was achieved by combined use of Random Forest and Gradient Boosting and use of diverse learning strategies. In addition, the importance of the temporal and environmental features extracted was emphasized by SHAP analysis as a contribution toward adding interpretability and strength.

The simplification of yield variability and the decision boundaries of the model is necessary for policy applications. However, the ambiguity of classification around a threshold and the gradations of yield performance cannot be captured in a binary framework. In principle, other model formulations, like multi-class classification (e.g. low/medium/high), could preserve finer-resolution yield distinctions, but this comes with higher model complexity and lower interpretability for nontechnical users. Of all the thresholds, the median threshold simplifies the model the most while also providing the most agronomically relevant outcome for forecasting at a regional scale.

Although its accuracy is very high there are still difficulties. Environmental variability may be one of the factors that are dangerous. In case of severe meteorological conditions or alterations of the soil condition can cause the models to become inaccurate. Relying on any type of historical information implies its own biases and cannot readily incorporate climate change as an issue. The data were moderate in terms of class distribution, (52% high yield/48% low yield), a situation where there is the least concern of bias; however, there is underrepresentation of events of extreme yields and this is likely to constrain the performance of the model to anomalous climate conditions. The model was trained on data in the U.S. Corn Belt and cannot be directly generalized to other regions that produce corn. Agronomic practices, Genotypes, and climatic conditions vary substantially across geographies. Future Work should utilize international datasets and make use of transfer learning or Domain adaptation frameworks for cross-regional applicability. Computationally, Ensembling techniques generally improve performance, but are more resource-intensive, which limits accessibility for small farms or institutions.

The feature inclusion of futures price variance is a point to be considered with care: it greatly enhanced the performance of the models; however, market prices already incorporate expected yield outcomes and weather forecasts, which may therefore introduce circularity in the reasoning. In the ablation study, removing market features decreased the accuracy by only 1.8%, which may indicate that agronomic and environmental factors are responsible for most of the predictability. We recommend the variant of the model without financial predictors for those applications where strict causal inference or out-of-sample generalization to non-market contexts is needed.

This article highlights the opportunities of machine learning in agricultural forecasting, especially in such methods associated with ensembles and explainability measures like SHAP. It mentions key issues that should be focused on like the variability of the climate, feature selection and historic dependence. Further environmental variables should be used in the future in creating more refined model architectures, and the out-of-sample validation should be done in large scale to get results that can be used in real life.

## Conclusion & future improvements

This study develops an interpretable ensemble machine learning model to predict county level(U.S) corn yield that will inform the planning and policy formulation of regional agriculture and take into consideration the spatial limitations inherent with aggregate information in as much as they can be applied to the field level. This study proposes that ensemble methods RandomForestClassifier and GradientBoostingClassifier are indeed very helpful to predicting whether a corn yield will fall above a threshold. For this, the model with these two classifiers is highly rated with respect to performance metrics such as accuracy, precision, recall, F1, and AUC. In this regard, the model’s robustness and generalizability is also confirmed with respect to confusion matrices, ROC curves, and accuracy scores. Moreover, the SHAP visualizations contribute to a better understanding of the model output with respect to the key explanatory feature. This exceptional performance means this model can be used as an aid to agricultural planners and managers, providing corn growers, policy makers, and agribusinesses with the means to forecast and identify seasons with low and high yield. In this aspect, the model could categorize various degrees of yield and it also assisted in strategies of crop management, resource optimization and prediction of food supply towards improved decisions in agriculture.

Further enhancements can involve spatial visualization of county-level predictions of observed vs. predicted yield maps and probability distribution across the U.S. Corn Belt, which would help to identify obscure patterns of geographical performance and accuracy of the regional model.The results identify some possible areas of improvement: the introduction of more agricultural, meteorological, and soil variables as well as the use of more advanced feature extraction methods to uncover hidden patterns in the crop-environmental relationship. Systematic hyperparameter tuning should provide the best performance for a set of tunable parameters. Exploring alternative models, such as neural networks, can capture more predictive patterns. It will need the integration of multi-regional datasets while considering domain adaptation frameworks because of the geographical variation that normally exists in crop management and other environmental conditions.

## Data Availability

The datasets used and analysed during the current study are available in the Kaggle repository, https://www.kaggle.com/datasets/guillemservera/grains-and-cereals-futures

## References

[1] Food and Agriculture Organization. The state of food security and nutrition in the world. *FAO* (2022).

[2] United States Department of Agriculture. World agricultural supply and demand estimates. *USDA* (2021).

[3] Ray, D. K., Gerber, J. S., MacDonald, G. K. & West, P. C. Climate variation explains a third of global crop yield variability. *Nat. Commun.* **6**, 5989 (2015). 25609225 10.1038/ncomms6989 PMC4354156

[4] Johnson, L. Sustainable agricultural practices and future trends. *Sustain. Dev.* **31**, 289–302 (2023).

[5] Karali, B. & Power, G. J. Short-and long-run determinants of commodity price volatility. *Am. J. Agric. Econ.* **95**, 724–738 (2013).

[6] Dhaliwal, D. S. & Williams, M. M. Sweet corn yield prediction using machine learning models and field-level data. *Precis. Agric.* **25**, 51–64 (2024).

[7] Khaki, S. & Wang, L. Crop yield prediction using deep neural networks. *Front. Plant Sci.* **10**, 621 (2019). 31191564 10.3389/fpls.2019.00621 PMC6540942

[8] Zhang, Y. Improving forecasting accuracy using LSTM networks. *J. Financ. Data Sci.* **12**, 78–89 (2021).

[9] Wang, L. Forecasting crop yields: The role of climate variables. *Environ. Sci. Policy* **102**, 55–66 (2020).

[10] Terliksiz, A. S. & Altilar, D. T. Impact of large kernel size on yield prediction: A case study of corn yield prediction with sedla in the US Corn Belt. *Environ. Res. Commun.* **6**, 025011 (2024).

[11] Lundberg, S. M. & Lee, S.-I. A unified approach to interpreting model predictions. *arXiv* (2017).

[12] Yang, X., Hua, Z., Li, L., Huo, X. & Zhao, Z. Multi-source information fusion-driven corn yield prediction using the random forest from the perspective of agricultural and forestry economic management. *Sci. Rep.* **14**, 4052 (2024). 38374339 10.1038/s41598-024-54354-9 PMC11325042

[13] Kussul, N. et al. Crop yield prediction with deep learning using remote sensing data. *J. Appl. Remote Sens.* **12**, 45–56 (2018).

[14] Zhang, H., Zheng, X. & Liu, S. Hybrid deep learning models for climate impacted crop yield prediction using ann, tabnet, and xgboost. *Clim.Sci.J.* **10**, 243–260 (2022).

[15] Bantchina, B. B. et al. Corn yield prediction in site-specific management zones using proximal soil sensing, remote sensing, and machine learning approach. *Comput. Electron. Agric.* **225**, 109329 (2024).

[16] Friedman, J. Greedy function approximation: A gradient boosting machine. *Ann. Stat.* **29**, 1189–1232 (2001).

[17] Rashid, M. Ensemble learning in forecasting: A review. *Forecasting* **3**, 12–24 (2020).

[18] Figueiredo, M. Predicting corn prices using machine learning models. *J. Appl. Econ.* **48**, 435–450 (2020).

[19] Sajid, S. S., Shahhosseini, M., Huber, I., Hu, G. & Archontoulis, S. V. County-scale crop yield prediction by integrating crop simulation with machine learning models. *Front. Plant Sci.* **13**, 1000224 (2022). 36518505 10.3389/fpls.2022.1000224 PMC9742473

[20] Schwalbert, R. et al. Mid-season county-level corn yield forecast for US Corn Belt integrating satellite imagery and weather variables. *Crop Sci.* **60**, 739–750 (2020).

[21] Ye, S., Cao, P. & Lu, C. Annual time-series 1 km maps of crop area and types in the conterminous US (CropAT-US): Cropping diversity changes during 1850–2021. *Earth Syst. Sci. Data* **16**, 3453–3470 (2024).

[22] USDA National Agricultural Statistics Service. Crop production 2023 summary. https://data.nass.usda.gov/Statistics_by_State/Delaware/Publications/Current_News_Release/2024/Jan2024-Crop-Production.pdf (2024).

[23] Kaya, E. & Ceylan, R. F. ARIMA and GARCH models for agricultural yield prediction. *Agric. Econ. Rev.* **20**, 123–135 (2019).

[24] Yao, Q. et al. Garch models in agricultural market volatility forecasting. *Journal of Agricultural Economics* **71**, 315–328 (2020).

[25] Zhang, M. & Liu, F. Time-series forecasting of corn yield using deep learning models. *J. Agric. Sci.* **30**, 45–60 (2023).

[26] Wimalasuriya, D. et al. Comparing statistical and machine learning models for corn yield prediction. *J. Agric. Sci.* **28**, 455–478 (2019).

[27] Li, F. & Wang, M. Limitations of traditional statistical models in agricultural forecasting. *J. Clim. Chang. Agric.* **10**, 120–135 (2022).

[28] Kumar, C., Dhillon, J., Huang, Y. & Reddy, K. N. Explainable machine learning models for corn yield prediction using uav multispectral data. *Available at SSRN 4744740* (2024).

[29] Jeong, J. H. et al. Random forest for corn yield prediction using multi-source data. *Comput. Electron. Agric.* **187**, 106263 (2021).

[30] Jang, C. et al. Integrating plant morphological traits with remote-sensed multispectral imageries for accurate corn grain yield prediction. *PLoS One* **19**, e0297027 (2024). 38564609 10.1371/journal.pone.0297027 PMC10986971

[31] Zhao, X. et al. Boosting algorithms in corn yield prediction: A gradient boosting machine approach. *Agric. Syst.* **188**, 103036 (2021).

[32] Meghraoui, K., Sebari, I., Pilz, J., Ait El Kadi, K. & Bensiali, S. Applied deep learning-based crop yield prediction: A systematic analysis of current developments and potential challenges. *Technologies* **12**, 43 (2024).

[33] Oliveira, G. et al. Random forest modeling for improving corn yield prediction: A case study integrating climate data. *Agron. J.* **114**, 655–666 (2022).

[34] Jeong, J. H. et al. Random forests for global and regional crop yield predictions. *PLoS One* **11**, e0156571 (2016). 27257967 10.1371/journal.pone.0156571 PMC4892571

[35] Shahhosseini, M., Hu, G., Huber, I. & Archontoulis, S. V. Coupling machine learning and crop modeling improves crop yield prediction in the US corn belt. *Sci. Rep.* **11**, 1606 (2021). 33452349 10.1038/s41598-020-80820-1 PMC7810832

[36] Maimaitijiang, M. et al. Using convolutional neural networks and multispectral data for crop yield prediction. *Remote. Sens.* **12**, 172 (2020).

[37] Kussul, N., Lavreniuk, M., Skakun, S. & Shelestov, A. Deep learning classification of land cover and crop types using remote sensing data. *IEEE Geosci. Remote Sens. Lett.* **14**, 778–782 (2017).

[38] You, J., Li, X., Low, M., Lobell, D. & Ermon, S. Deep gaussian process for crop yield prediction based on remote sensing data. In *Proceedings of the AAAI conference on artificial intelligence*, vol. 31 (2017).

[39] Jiang, X. et al. A hybrid cnn-lstm model for predicting crop yields. *Comput. Electron. Agric.* **198**, 107116 (2022).

[40] Khaki, S., Wang, L. & Archontoulis, S. V. A cnn-rnn framework for crop yield prediction. *Front. Plant Sci.* **10**, 1750 (2020). 32038699 10.3389/fpls.2019.01750 PMC6993602

[41] Lobell, D. B., Thau, D., Seifert, C., Engle, E. & Little, B. A scalable satellite-based crop yield mapper. *Remote Sens. Environ.* **164**, 324–333 (2015).

[42] Guan, K. et al. The shared and unique values of optical, fluorescence, thermal and microwave satellite data for estimating large-scale crop yields. *Remote Sens. Environ.* **199**, 333–349 (2017).

[43] Campos-Taberner, M. et al. Drone-based imaging and cnn for improving corn yield predictions. *Agric. Syst.* **193**, 103219 (2022).

[44] Smith, J., Lee, A. & Williams, M. Predicting corn yield using machine learning: An evaluation of different approaches. *J. Agric. Data Sci.* **5**, 145–160 (2021).

[45] Breiman, L. Random forests. *Mach. Learn.* **45**, 5–32 (2001).

[46] Lundberg, S. M. & Lee, S.-I. A unified approach to interpreting model predictions. *Adv. Neural Inf. Process. Syst.* **30**, 4765–4774 (2017).

[47] United States Department of Agriculture. Usda quick stats database. https://quickstats.nass.usda.gov/ (2023). Accessed: 2025-08-19.

[48] National Oceanic and Atmospheric Administration. Noaa climate data. https://www.noaa.gov/ (2022). Accessed: 2025-08-19.

[49] Chicago Mercantile Exchange. Grains and cereals futures dataset. https://www.kaggle.com/datasets/guillemservera/grains-and-cereals-futures (2023). Accessed: 2025-08-19.

[50] NASA POWER Project. Nasa power climate data. https://power.larc.nasa.gov/ (2023). Accessed: 2025-08-19.

[51] ECMWF. Era5 reanalysis data. https://www.ecmwf.int/en/forecasts/datasets/reanalysis-datasets/era5 (2023). Accessed: 2025-08-19.

[52] SoilGrids. Global soil information system. https://soilgrids.org/ (2023). Accessed: 2025-08-19.

[53] NASA MODIS. Moderate resolution imaging spectroradiometer (modis). https://modis.gsfc.nasa.gov/ (2023). Accessed: 2025-08-19.

[54] European Space Agency. Sentinel-2. https://www.esa.int/Applications/Observing_the_Earth/Copernicus/Sentinel-2 (2025). Accessed: 2025-08-10.

[55] Khramtsov, R. R. & Koposov, S. E. Machine learning methods for astronomical classification tasks and crop yield prediction using catboost, xgboost, and random forest. *Astron. J.* **158**, 1–10 (2020).

[56] Espejo, G., Ventura, S. & Herrera, F. A survey on the application of ensemble learning for crop yield prediction. *Inf. Fusion* **16**, 3–19 (2010).

